# The Adjustment of Anterior Forebrain Pathway (AFP) to Birdsong Is Phased during Song Learning and Maintenance

**DOI:** 10.1155/2020/6647389

**Published:** 2020-12-16

**Authors:** Jie Zang, Shenquan Liu

**Affiliations:** School of Mathematics, South China University of Technology, Guangzhou 510640, China

## Abstract

Anterior forebrain pathway (AFP), a basal ganglia-dorsal forebrain circuit, significantly impacts birdsong, specifically in juvenile or deaf birds. Despite many physiological experiments supporting AFP's role in song production, the mechanism underlying it remains poorly understood. Using a computational model of the anterior forebrain pathway and song premotor pathway, we examined the dynamic process and exact role of AFP during song learning and distorted auditory feedback (DAF). Our simulation suggests that AFP can adjust the premotor pathway structure and syllables based on its delayed input to the robust nucleus of the archistriatum (RA). It is also indicated that the adjustment to the synaptic conductance in the song premotor pathway has two phases: normal phases where the adjustment decreases with an increasing number of trials and abnormal phases where the adjustment remains stable or even increases. These two phases alternate and impel a specific effect on birdsong based on AFP's specific structures, which may be associated with auditory feedback. Furthermore, our model captured some characteristics shown in birdsong experiments, such as similarities in pitch, intensity, and duration to real birds and the highly abnormal features of syllables during DAF.

## 1. Introduction

Birdsong is a complex learned behavior based on neural circuits and premotor functions. The neural structure is well-delineated [1] and similar to that for humans [2], leading to a great interest spreading among researchers. Through a series of electrophysiological experiments, researchers have demonstrated that song production mainly involves the song premotor pathway from the nucleus High Vocal Center (HVC) to the robust nucleus of the archistriatum (RA). They also found that song learning involves the anterior forebrain pathway (AFP), a basal ganglia-dorsal forebrain circuit composed of nucleus Area X, the dorsolateral thalamus (DLM), and the lateral part of the magnocellular nucleus of the anterior neostriatum (LMAN) [3]. In particular, AFP's neurons in juvenile or deaf birds become more active than adults and adjust syllables for a specific purpose, such as learning.

The observations by Brainard [4] demonstrated that juvenile birds learn songs based on AFP, and the AFP lesions can damage their normal learning process. The lesions make the completion of song learning (called crystallization) occur prematurely and result in songs with highly abnormal features [5]. However, they do not affect adult birds since birdsong has crystallized. On the other hand, song degradation happens with adult birds deafened and breaks with lesions of LMAN [6]. These phenomena show that AFP has the ability to adjust birdsong and may affect the premotor pathway structure, which is closely related to syllables [7].

Due to these observed phenomena, the adjustment of AFP to birdsong during song learning and maintenance has attracted researchers' attention. In 2004, Brainard [4] discussed the potential instructive and permissive functions of AFP in vocal plasticity. For song learning in juvenile birds, Kojima and Doupe [8] found that AFP neurons without tutor song exposure had highly tuned responses to bird's own song based on recording physiological data. Furthermore, Hamaguchi et al. [5] showed that deafening-induced changes to HVC synapses require intact AFP output through in vivo multiphoton imaging. Despite important progress in identifying AFP's role in vocal plasticity, there is no good understanding of these experiments' neural mechanisms. Therefore, it is effective and necessary to use neural model theory to study AFP's effect during these processes.

In this study, we extended the birdsong premotor pathway model introduced by Abarbanel et al. [9] to an AFP-mediated model, focusing on the role of AFP during song learning and DAF. The model's modification includes three aspects: (1) HVC_X_ neurons were introduced in HVC to control AFP and can be regulated according to environmental and physiologic factors. Imaging studies have shown that deafening causes the dendritic spines of HVC_X_ neurons to contract within 12-48 hours, predicting the syllable degradation process [10]. (2) The fixed synaptic connection between HVC and RA was modified to be random and adjustable, resulting in synaptic plasticity of the premotor pathway, which provides the basis for AFP regulation. (3) An AFP model [11] was embedded in the song premotor pathway, with its network structure expanded based on some studies on nucleus DLM and Area X [12, 13]. Besides, we hypothesized that the structure of the nucleus in HVC and AFP had been well regulated by their superior nuclei.

For gaining insight into the control and maintenance functions of AFP on song syllables, we trained the extended model to simulate two different syllable adjustment processes: the learning process in young birds and the syllable distortion process in deaf birds. The simulation results successfully reproduced these two adjustment processes and quantitatively demonstrated AFP's functions. They showed that AFP's adjustment is phased, with normal phases and abnormal phases. During most of the adjustment process called normal phases, AFP's adjustment amount and adjustment rate decreased with more trials. However, in other intervals called abnormal phases, they remained stable or even increased. Our simulation explained the neural mechanism underlying song production and adjustment, indicating the important role of neural plasticity during birdsong learning and maintenance.

## 2. Methods of Song Premotor Pathway

**Figure 1:**
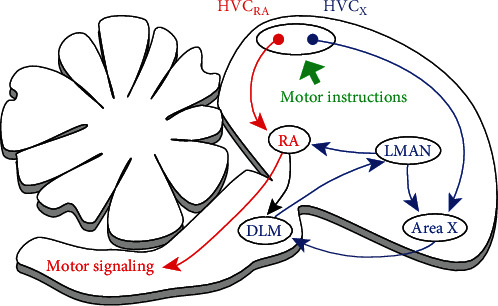
Neural structure of birdsong neural circuit. The red arrows indicate the song premotor pathway from HVC to RA. Similarly, the blue arrows indicate the anterior forebrain pathway (AFP) consisted of areas X, DLM, and LMAN.

The neural circuit of the song premotor pathway is shown in Figure 1, accompanied by the anterior forebrain pathway (AFP) consisted of areas X, DLM, and LMAN. In the song premotor pathway, HVC excitatory neurons fire sparsely [6] during song production to control moments and sequences of syllables, minimum units of a song [14]. The output of HVC is directly and indirectly via AFP sent to RA, where excitatory neurons control the syrinx muscles and respiratory action [15]. RA projection neurons have different activity patterns [16], which are perceived as causes of different syllables. In fact, Henry has studied the relationship between song features and nerve structure, supporting that intrinsic the circuit within RA may greatly influence the features of birdsong [9]. Also, AFP participates in the song learning and maintenance as a processor between HVC and RA. Compared to that from HVC, AFP's stimulation is sent to RA with a delay of 50 ± 10 ms.

Here, we make some improvements on the song premotor pathway model proposed by Abarbanel et al. [9]: introducing HVC_X_ neurons to control AFP, regulating the network structure of HVC to adjust for the connecting with AFP, and improving the synaptic connection between HVC and RA to be random and plastic.

### 2.1. Nucleus HVC

Nucleus HVC contains several types of neurons, including projection neurons (HVC_RA_ and HVC_X_ neurons) that project to RA and Area X and interneurons (HVC_I_ neurons) that project locally within the HVC [17]. These different neuron types have different morphologies, and few projection neurons project to both RA and area X [18].

We construct 20 HVC projection neurons and 2 inhibitory neurons to simulate HVC in the form of a feed-forward model. During each syllable, projection neurons fire sparsely while inhibition neurons receive input from them and inhibit them in turn. Based on the HH equation, the membrane potential of the *j*th HVC_RA_ neuron *V*_HR_^*j*^(*t*) (*j* = 1, 2, ⋯, 10) is given by

(1) $$\begin{array}{c} C_{M}\frac{{dV}_{\text{HR}}^{j}\left(t\right)}{dt}=I_{\text{HH}\_\text{HVC}}+I_{\text{HR}‐\text{HR}}+I_{\text{HX}‐\text{HR}}+I_{\text{HI}‐\text{HR}}+I_{\text{DC}\_\text{HR}}, \end{array}$$

where HR, HX, and HI represent neurons HVC_RA_, HVC_X_, and HVC_I_, respectively; *I*_DC_HR_ indicates the direct current stimulation to HVC_RA_; and *I*_HR_HR_, *I*_HX_HR_, and *I*_HI_HR_ represent the input from other HVC neurons. When *j* = 1, *I*_HR_HR_, and *I*_HX_HR_ are ignored, *I*_HH_HVC_, including leakage, sodium, and potassium currents, is given by

(2) $$\begin{array}{c} I_{\text{HH}\_\text{HVC}}=g_{L}\left[E_{L}-V_{\text{HR}}^{j}\left(t\right)\right]+g_{\text{Na}}m_{j}{\left(t\right)}^{3}h_{j}\left(t\right)\left[E_{\text{Na}}-V_{\text{HR}}^{j}\left(t\right)\right]+g_{K}n_{j}^{4}\left[E_{K}-V_{\text{HR}}^{j}\left(t\right)\right], \end{array}$$

where *g*_L_, *g*_Na_, and *g*_K_ are the maximum conductance of Na, K, and leakage current. The activation and deactivation variables *m*_*j*_(*t*), *h*_*j*_(*t*), and *n*_*j*_(*t*) satisfy the following first-order kinetic equations,

(3) $$\begin{array}{c} \frac{{dX}_{j}\left(t\right)}{dt}=\alpha_{x}\left[V_{\text{HR}}^{j}\left(t\right)\right]\left[1-X_{j}\left(t\right)\right]-\beta_{x}\left[V_{\text{HR}}^{j}\left(t\right)\right]X_{j}\left(t\right), \end{array}$$

where *X*_*j*_(*t*) represents *m*_*j*_(*t*), *h*_*j*_(*t*), and *n*_*j*_(*t*). The corresponding parameters *α*_*x*_ and *β*_*x*_ are

(4) $$\begin{array}{c} \begin{array}{c} \alpha_{m}\left(V\right)=\frac{-3.2\left(V+50\right)}{e^{-\left(V+50\right)/4}-1},\\ \beta_{m}\left(V\right)=\frac{2.8\left(V+25\right)}{e^{\left(V+25\right)/5}-1},\\ \alpha_{h}\left(V\right)=1.28e^{-\left(V+48\right)/18},\\ \beta_{h}\left(V\right)=\frac{40}{e^{-\left(V+25\right)/5}+1},\\ \alpha_{n}\left(V\right)=\frac{-0.32\left(V+50\right)}{e^{-\left(V+50\right)/5}-1},\\ \beta_{n}\left(V\right)=5e^{-\left(55+V\right)/40}. \end{array} \end{array}$$

Inputs from the neurons inside HVC are represented by

(5) IHR‐HR=gHR‐HRSGVHRj−1tERevE−VHRj,IHX‐HR=gHX‐HRSGVHXj−1tERevE−VHRj,IHI‐HR=gHI‐HRSGVHI1tERevI−VHRj,

where the function *S*_*G*_ [*V*_pre_ (*t*)] represents the proportion of postsynaptic GABA receptor channels opened, and it satisfies first-order kinetics,

(6) $$\begin{array}{c} \frac{{dS}_{G}\left[V_{\text{pre}}\left(t\right)\right]}{dt}=0.15\frac{1-S_{G}\left[V_{\text{pre}}\left(t\right)\right]}{1+e^{-V_{\text{pre}}\left(t\right)+10}}-0.2275S_{G}\left[V_{\text{pre}}\left(t\right)\right]. \end{array}$$

The membrane potential of the *j*th HVC_X_ neuron *V*_HX_^*j*^(*t*) can be similarly given by

(7) $$\begin{array}{c} C_{M}\frac{{dV}_{\text{HX}}^{j}\left(t\right)}{dt}=I_{\text{HH}\_\text{HVC}}+I_{\text{HR}‐\text{HX}}+I_{\text{HX}‐\text{HX}}+I_{\text{HI}‐\text{HX}}+I_{\text{DC}\_\text{HX}}, \end{array}$$

where *V*_HX_^*j*^(*t*) is inhibited by HVC inhibitory neurons and other related inputs are similar to that of HVC_RA_ neurons.

In addition, the membrane voltage *V*_HI_^*i*^(*t*) (*i* = 1, 2) of the two inhibitory neurons satisfies the HH equations,

(8) $$\begin{array}{c} \begin{array}{c} C_{M}\frac{{dV}_{\text{HI}}^{1}\left(t\right)}{dt}=I_{\text{HH}‐\text{HVC}}\left(t,V_{\text{HI}}^{1}\left(t\right)\right)+I_{\text{HR}‐\text{HI}},\\ C_{M}\frac{{dV}_{\text{HI}}^{2}\left(t\right)}{dt}=I_{\text{HH}‐\text{HVC}}\left(t,V_{\text{HI}}^{2}\left(t\right)\right)+I_{\text{HX}‐\text{HI}}, \end{array} \end{array}$$

where *I*_HR−HI_ and *I*_HX−HI_ represent inputs from projection neurons to inhibitory neurons, expressed as follows:

(9) IHR‐HI=∑j=110gHR‐HISGVHRjtERevE−VHI1,IHX‐HI=∑j=110gHX‐HISGVHXjtERevE−VHI2.

The parameters used for HVC neurons are *C*_M_ = 1 *μ*F/cm^2^, *g*_Na_ = 215 mS/cm^2^, *g*_K_ = 43 mS/cm^2^, *g*_L_ = 0.83 mS/cm^2^, *E*_Na_ = −50 mV, *E*_K_ = −95 mV, *E*_L_ = −65 mV, *g*_HR−HR_ = g_HX−HX_ = 12.7 mS/cm^2^, *g*_HX−HR_ = *g*_HX−HR_ = 7.7 mS/cm^2^, *g*_HI−HR_ = *g*_HI−HX_ = 7.5 mS/cm^2^, *g*_HR−HI_ = *g*_HX−HI_ = 3.5 mS/cm^2^, *E*_RevE_ = 0 mV, and *E*_RevI_ = −80 mV. When the neural circuit is at rest, *I*_DC_HR_ = *I*_DC_HR_ = 0 *μ*A/cm^2^.

### 2.2. Nucleus RA

**Figure 2:**
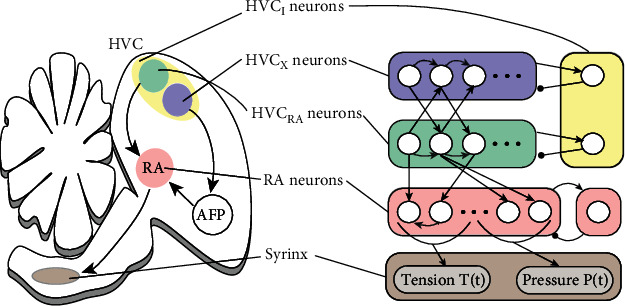
Neural structure of the song premotor pathway. HVC contains several types of neurons, including projection neurons (HVC_X_ and HVC_RA_) that project to RA and area X and interneurons HVC_I_ that project locally within the HVC. HVC_X_ and HVC_RA_ act in the form of a feed-forward model to fire sparsely. Besides, RA has two types of neurons: inhibitory neurons and projection neurons. Inhibitory neurons are used to simulate the local inhibition phenomenon in RA, while projection neurons are stimulated by HVC and LMAN and divided into two categories. One part controls the muscle of syrinx through “spring constant” *T*(*t*), and the other part controls the respiratory system through driving pressure *P*(*t*). Arrow lines represent excitatory projections, while circle-headed lines represent inhibitory projections.

As shown in Figure 2, there are inhibitory and excitatory neurons in RA. Inhibitory neurons simulate the local inhibition phenomenon in RA, while excitatory neurons receive input from HVC and LMAN and control syrinx to produce birdsong. Some RA neurons control the muscle of syrinx through the “spring constant” *T*(*t*), and others control the respiratory system through the driving pressure *P*(*t*). Here, we use 5 RA projection neurons to represent each part. The membrane voltage *V*_RP_^*n*^(*t*) of the *n*th projection neuron satisfies:

(10) $$\begin{array}{c} C_{M}\frac{{dV}_{\text{RP}}^{n}\left(t\right)}{dt}=I_{\text{HH}\_\text{RA}}+I_{\text{HR}‐\text{RP}}+I_{\text{LMAN}‐\text{RP}}+I_{\text{RI}‐\text{RP}}+I_{\text{RP}‐\text{RP}}+I_{\text{DC}\_\text{RP}}, \end{array}$$

where RP, RI stands for RA excitatory and inhibitory neurons separately; *I*_DC_RP_ indicates direct current stimulation to projection neurons; *I*_RI−RP_ and *I*_RP−RP_ represent the input of other neurons in RA; and *I*_HR−RP_ and *I*_LMAN−RP_ represent the input from HVC and LMAN. These inputs are given by

(11) IHR‐RP=∑j=110ΓHR‐RPj,ngHR‐RPSGVHRjtERevE−VRPn,ILMAN‐RP=∑j=110ΓLMAN‐RPj,ngLMAN‐RPSGVLMAN‐RPjtERevE−VRPn,IRP‐RP=∑j=1,j≠n10ΓRP‐RPj,ngRP‐RPSGVRPjtERevE−VRPn,IRI‐RP=gRI‐RPSGVRItERevI−VRPn,

where *g*_HR−RP_ and *g*_HR−LMAN_ represent the maximum synaptic conductance from HVC_RA_ and LMAN to RA projection neurons, respectively; *g*_HR−HR_ represents the maximum synaptic conductance between RA projection neurons; *Г*_HR−RP_, *Г*_LMAN−RP_, and *Г*_RP−RP_ are 10 × 10 matrices, and they determine the strength of synaptic connections. *Г*_LMAN−RP_ is an identity matrix, while *Г*_HR−RP_ and *Г*_RP−RP_ are random matrices where each element in matrices takes a random number between [0.01, 1].

Besides, RA inhibition neuron receives input from HVC_RA_ and RA projection neurons, whose membrane voltage satisfies

(12) $$\begin{array}{c} C_{M}\frac{{dV}_{\text{RI}}\left(t\right)}{dt}=I_{\text{HH}\_\text{RA}}+I_{\text{HR}‐\text{RI}}+I_{\text{RP}‐\text{RI}}+I_{\text{DC}\_\text{RI}}, \end{array}$$

where inputs from RA projection neurons and HVC_RA_ neurons are given by

(13) IHR‐RI=∑j=110gHR‐RISGVHRjtERevE−VRI,IRP‐RI=∑j=110gRP‐RISGVRPjtERevE−VRI.

The following are parameters used for RA neurons: *C*_*M*_ = 1 *μ*F/cm^2^, *g*_Na_ = 215 mS/cm^2^, *g*_K_ = 43 mS/cm^2^, *g*_L_ = 0.83 mS/cm^2^, *E*_Na_ = −50 mV, *E*_K_ = −95 mV, *E*_L_ = −65 mV, *g*_HR−RP_ = 18.62 mS/cm^2^, *g*_LMAN−RP_ = 7.7 mS/cm^2^, *g*_RP−RP_ = 0.35 mS/cm^2^, *g*_RI−RP_ = 50 mS/cm^2^, *g*_HR−RI_ = 0.5 mS/cm^2^, *g*_RP−RI_ = 0.75 mS/cm^2^, *E*_RevE_ = 0 mV, *E*_RevI_ = −80 mV, *I*_DC_RP_ = 3.5 mS/cm^2^, and *I*_DC_RI_ = 0 mS/cm^2^.

## 3. Methods of Anterior Forebrain Pathway

**Figure 3:**
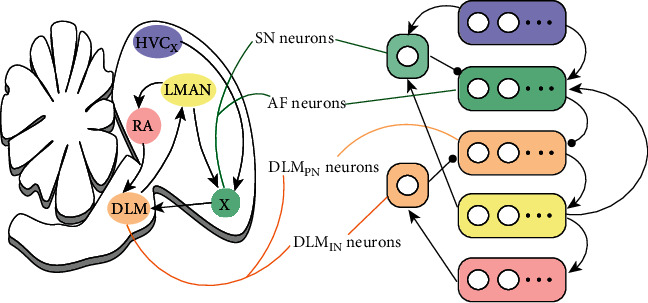
Neural structure of the anterior forebrain pathway (AFP) consisted of Area X, DLM, and LMAN. There are spiny neurons (SN) and fast-spiking neurons (AF) in Area X, stimulated by HVC_X_ neurons. AF neurons also get inputs from SN neurons and LMAN while simultaneously inhibiting DLM projection neurons (DLM_PN_). DLM_PN_ neurons are under local inhibition caused by the DLM inhibitory neuron (DLM_IN_). They stimulate LMAN, which finally sends delayed input to RA. Inhibitory neurons, SN and DLM_IN_, are activated by LMAN and RA, respectively. Arrow lines represent excitatory projections, while circle-headed lines represent inhibitory projections.

As shown in Figure 3, AFP consists of Area X, DLM, and LMAN. Based on its internal structure, AFP processes information from HVC_X_ and sends input to RA with a delay of 50 ± 10 ms compared to HVC_RA_. For juvenile birds, AFP helps them learn birdsong. Any lesion of AFP, such as bilateral lesions in area X or LMAN, will disrupt their song development process [19]. Moreover, AFP injuries can prevent song degradation caused by deafening. To simulate these phenomena, we expanded the structure of the AFP model introduced by Abarbanel et al. [11] and embedded the model to the song premotor pathway.

### 3.1. Nucleus Area X

Stimulated by HVC and LMAN, Area X has excitatory projections to DLM. As shown in Figure 3, there are spiny neurons (SN) and aspiny fast-spiking neurons (AF) in Area X [20]. During periods of silence (nonsinging), SN neurons are in the polarization state, while AF neurons are in the oscillatory regime at 15-30 Hz.

In our model, the SN neuron receives input from HVC and LMAN. Its membrane voltage *V*_SN_ is given by:

(14) $$\begin{array}{c} C_{M}\frac{{dV}_{\text{SN}}\left(t\right)}{dt}=I_{\text{HH}\_\text{AFP}}+I_{\text{HX}‐\text{SN}}+I_{\text{LMAN}‐\text{SN}}+I_{\text{DC}\_\text{SN}}, \end{array}$$

where *I*_DC−SN_ is the direct current stimulation and *I*_HH___AFP_ is similar to *I*_HH___HVC_. *I*_HX−SN_ and *I*_LMAN−SN_ indicating the input from HVC_X_ and LMAN neurons are expressed as

(15) IHX‐SN=gHX‐SN∑j=110SAVHXjtERevE−VSN,ILMAN‐SN=gLMAN‐SN∑j=110SAVLMANjtERevE−VSN.

Here, the function *S*_*A*_[*V*_pre_(*t*)] represents the proportion of open postsynaptic AMPA receptor channels. It satisfies first-order dynamics,

(16) $$\begin{array}{c} \frac{{dS}_{A}\left[V_{\text{pre}}\left(t\right)\right]}{dt}=5\left\{1+\tan h\left[120\left(V_{\text{pre}}-0.1\right)\right]\right\}-10S_{A}\left[V_{\text{pre}}\left(t\right)\right]. \end{array}$$

In addition, 10 AF neurons receive the input from HVC_X_ to Area X. Their membrane voltage *V*_AF_^*j*^(*t*) (*j* = 1, 2, ⋯, 10) of the *j*th AF neuron is given by

(17) $$\begin{array}{c} C_{M}\frac{{dV}_{\text{AF}}^{j}\left(t\right)}{dt}=I_{\text{HH}\_\text{AFP}}\left(t,V_{\text{SN}}\left(t\right)\right)+I_{\text{HX}‐\text{AF}}+I_{\text{LMAN}‐\text{AF}}+I_{\text{SN}‐\text{AF}}+I_{\text{DC}\_\text{AF}}, \end{array}$$

where *I*_DC−AF_ represents the direct current stimulation to AF neurons, *I*_SN−AF_ represents the inhibition from SN, *I*_HX−AF_ represents the input from HVC_X_ neurons, and *I*_LMAN−AF_ represents the input from LMAN neurons. These currents are

(18) ISN‐AF=gSN‐AFSAVSNtERevI−VAFj,IHX‐AF=∑i=110ΓHX‐AFi,jgHX‐AFSAVHXitERevE−VAFj,ILMAN‐AF=∑i=1,i≠j10ΓLMAN‐AFi,jgLMAN‐AFSAVLMANitERevE−VAFj,

where *g*_HX−AF_ represents the maximum conductance of the synapse from HVC_X_ to the AF and *g*_LMAN−AF_ represents that from LMAN to the AF neuron. The matrices Γ_HX−AF_ and Γ_LMAN−AF_ are 10 × 10 matrices, which determine the strength of the corresponding synaptic connection.

The following are the parameters used for neurons in AFP: *C*_M_ = 1 *μ*F/cm^2^, *g*_Na_ = 20 mS/cm^2^, *g*_K_ = 6.2 mS/cm^2^, *g*_L_ = 0.03 mS/cm^2^, *E*_Na_ = 50 mV, *E*_K_ = −99 mV, *E*_L_ = −49.4 mV, *E*_RevE_ = 0 mV, and *E*_RevI_ = −80 mV. In Area X, *g*_HX−SN_ = 0.4 mS/cm^2^, *g*_LMAN−SN_ = 0.4 mS/cm^2^, *g*_SN−AF_ = 0.08 mS/cm^2^, *g*_HX−AF_ = 0.4 mS/cm^2^, *g*_LMAN−AF_ = 0.1 mS/cm^2^, *I*_DC−SN_ = −0.82 mS/cm^2^, and *I*_DC−AF_ = −0.42 mS/cm^2^.

### 3.2. Nucleus DLM

In DLM, inhibited by AF neurons and DLM inhibitory neurons, projection neurons activate LMAN. They include low threshold Ca^2+^ currents, *I*_*T*_, and *I*_*h*_, thus showing the characteristics of delayed activity.

We establish 10 DLM projection neurons, whose membrane potentials are given by

(19) $$\begin{array}{c} C_{M}\frac{{dV}_{\text{DP}}^{j}\left(t\right)}{dt}=I_{\text{HH}\_\text{AFP}}+I_{h}+I_{T}+I_{\text{AF}‐\text{DP}}+I_{\text{DI}‐\text{DP}}+I_{\text{DC}\_\text{DP}}, \end{array}$$

where DP stands for DLM projection neurons and DI stands for DLM inhibitory neurons. The currents from AF neurons (*I*_AF−DP_) and DLM inhibitory neuron (*I*_DI−DP_) are given by

(20) IAF‐DP=∑i=110ΓAF‐DPi,jgAF‐DPSVAFitERevI−VDPj,IDI‐DP=gDI‐DPSVDItERevI−VDPj,

where *g*_AF−DP_ represents the maximum synaptic conductance from AF to DLM projection neurons, and *g*_DI−DP_ represents that from DLM inhibitory neurons to DLM projection neurons. The matrix Γ_AF−DP_ is a 10 × 10 matrix, which determines the strength of the corresponding synaptic connection.

Besides, the low threshold Ca^2+^ currents, *I*_*T*_ and *I*_*h*_, have the following forms:

(21) $$\begin{array}{c} \begin{array}{c} I_{h}\left(t\right)=g_{h}m_{h}\left(t\right)\left[E_{h}-V_{\text{DP}}^{j}\left(t\right)\right],\\ I_{T}\left(t\right)=g_{T}m_{c}\left(t\right)h_{c}\left(t\right)\text{GHK}\left[V_{\text{DP}}^{j}\left(t\right)\right], \end{array} \end{array}$$

where the Goldman-Hodgkin-Katz expression is

(22) $$\begin{array}{c} \text{GHK}\left(V\right)=-V\frac{1-\left({\left[{\text{Ca}}^{2+}\right]}_{O}/{\left[{\text{Ca}}^{2+}\right]}_{i}\right)e^{-V/12.9}}{1-e^{-V/12.9}}. \end{array}$$

The activation and inactivation variables *U*(*t*) = *m*_*h*_(*t*), *m*_*c*_(*t*), *h*_*c*_(*t*) satisfy the first-order kinetic equations

(23) $$\begin{array}{c} \frac{dU\left(t\right)}{dt}=\frac{U_{0}\left(V_{\text{DP}}^{j}\left(t\right)\right)-U\left(t\right)}{\tau_{U}\left(V_{\text{DP}}^{j}\left(t\right)\right)}, \end{array}$$

and the expressions of *U*_0_ and *τ*_*U*_ are

(24) $$\begin{array}{c} \begin{array}{c} m_{c0}\left(V\right)=1/\left[1+e^{-\left(V+60\right)/6.2}\right],\\ \tau_{mc}\left(V\right)=0.612+1/\left[e^{-\left(V+131\right)/16.7}+e^{-\left(V+16.8\right)/12.9}\right],\\ h_{c0}\left(V\right)=1/\left[1+e^{\left(V+84\right)/4.03}\right],\\ \tau_{hc}\left(V\right)=28+e^{-\left(28.8+V\right)/10.2},\\ m_{h0}\left(V\right)=1/\left[1+e^{\left(V+75\right)/5.5}\right],\\ \tau_{h}\left(V\right)=0.612+1/\left[e^{-\left(V+131.6\right)/16.7}+e^{\left(V+16.8\right)/18.2}\right]. \end{array} \end{array}$$

Besides, DLM inhibition neuron membrane voltage is given by

(25) CMdVDItdt=IHH_AFP+IRA‐DI+IDC‐DI,IRA‐DI=gRA‐DI∑i=110SAVRAiERevE−VDI.

The following are the parameters used for DLM neurons: *g*_AF−DP_ = 0.4 mS/cm^2^, *g*_DI−DP_ = 4 mS/cm^2^, *g*_RA−DI_ = 1.5 mS/cm^2^, *g*_h_ = 0.045 mS/cm^2^, *g*_T_ = 3.775 × 10^−5^ mS/cm^2^, [Ca^2+^]/[Ca^2+^]_*i*_ = 4000, *I*_DC−DP_ = −3 mS/cm^2^, and *I*_DC−DI_ = −0.55 mS/cm^2^.

### 3.3. Nucleus LMAN

LMAN receives input from the DLM projection neuron and stimulates RA. We established 10 neuron models to simulate LMAN. The membrane voltage of the *j*th neuron is

(26) $$\begin{array}{c} C_{M}\frac{{dV}_{\text{LMAN}}^{j}\left(t\right)}{dt}=I_{\text{HH}\_\text{AFP}}+I_{\text{DP}‐\text{LMAN}}+I_{\text{DC}\_\text{LMAN}}, \end{array}$$

where the current *I*_DP−LMAN_ of the DLM projection neuron is given as

(27) IDP‐LMAN=∑i=110ΓDP‐LMANi,jgDP‐LMANSVDPitERevE−VLMANj.

*g*_DP−LMAN_ represents the maximum synaptic conductance from DLM projection neurons to LMAN neurons. The matrix Γ_DP−LMAN_ is a 10 × 10 matrix, which determines the strength of the corresponding synaptic connection.

It was suggested that the delayed stimulation from LMAN adjust the structure of the song premotor pathway to change the song [21]. Henry established a simple biophysical model of synaptic plasticity, including mixed receptors of NMDAR and AMPAR. In Henry's model, the synaptic plasticity is controlled by Δ*T* (time difference between inputs to RA from LMAN and HVC), which is used in this paper. In order to simplify our calculation process, we assume that the adjustment function controlled by Δ*T* satisfies

(28) $$\begin{array}{c} \frac{\Delta g_{\text{RA}}}{g_{\text{RA}}}=\left\{\begin{array}{c} 5.333\times 10^{-7}{\left(\Delta T\right)}^{3}-5.829\times 10^{-5}{\left(\Delta T\right)}^{2}-2.619\times 10^{-5}\left(\Delta T\right)+0.08211\;0<\Delta T<75\\ -0.0225e^{-x+75}\;75\leq \Delta T \end{array}\right., \end{array}$$

which can fit the results obtained by Henry well. We set the minimum value of conductance as 0.01 × *g*_max_ and the maximum value as *g*_max_ in the calculation. Besides, *g*_DP−LMAN_ = 0.4 mS/cm^2^ and *I*_DC−LMAN_ = −0.85 mS/cm^2^.

## 4. Methods of Syrinx

To present AFP's adjustment to song premotor pathway in the form of birdsong, we use a simplified vocal dynamic system [22] as the syrinx model to show syllables caused by RA. Based on the model proposed previously, the activity of RA is converted into two control parameters, *P*(*t*) and *T*(*t*), whose specific expressions are

(29) $$\begin{array}{c} \begin{array}{c} \frac{dT\left(t\right)}{dt}={\left\{\text{Pos}\left[\underset{a}{\sum }V_{a}^{T,\text{RA}}\left(t\right)-\theta \right]\right\}}^{7/4}-\frac{T\left(t\right)}{\tau_{\text{RA}}},\\ \frac{dP\left(t\right)}{dt}={\left\{\text{Pos}\left[\underset{a}{\sum }V_{a}^{P,\text{RA}}\left(t\right)-\theta \right]\right\}}^{7/4}-\frac{P\left(t\right)}{\tau_{\text{RA}}}, \end{array} \end{array}$$

where if *x* > 0 and Pos(*x*) = *x*; otherwise, Pos(*x*) = 0. The movement of the labia midpoint in the syrinx model is given by

(30) $$\begin{array}{c} \begin{array}{c} \frac{dx\left(t\right)}{dt}=y\left(t\right),\\ \frac{dy\left(t\right)}{dt}=-\left[\alpha_{1}T\left(t\right)+\alpha_{0}\right]x+Cx{\left(t\right)}^{2}y\left(t\right)+\left[\beta_{1}P\left(t\right)+\beta_{0}-b\right]y\left(t\right), \end{array} \end{array}$$

where *α*_1_ = 3.75 × 10^8^, *α*_0_ = 5.93 × 10^5^, *C* = 2 × 10^8^, *β*_1_ = 30, and *β*_0_ = 80 × 10^8^.

## 5. Results

### 5.1. Birdsong Production

**Figure 4:**
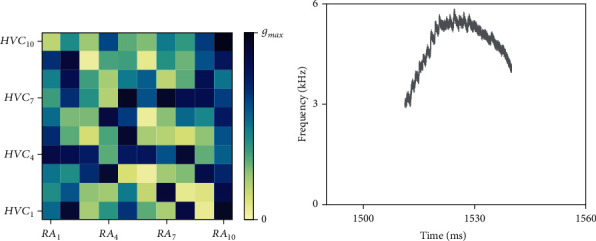
Intensity distribution of the synaptic conductance matrix and the corresponding syllable produced. (a) A random synaptic conductance matrix *g*_HR−RP_ × Γ_HR−RP_ with each element from 0 to *g*_HR−RP_. The square in (*i*, *j*) represents the synaptic conductance from HVC_i_ to RA_j_ with its color depth indicating the intensity of conductance. (b) The corresponding syllables lasting 27.3 ms with its basic frequency between 3 and 6 kHz.

**Figure 5:**
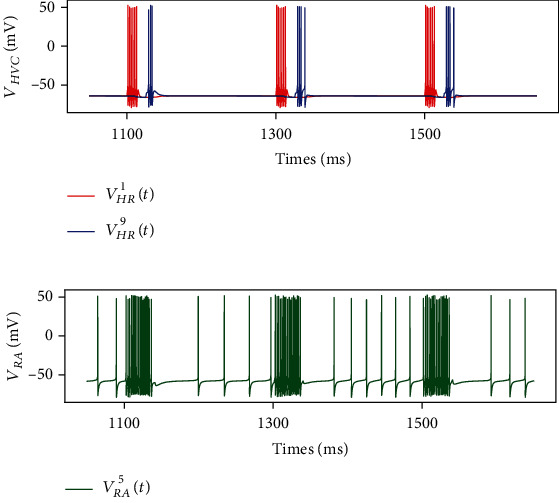
Membrane voltages of neurons in the song premotor pathway. Before the song, HVC_RA_ neurons are in the polarization state (a), and RA projection neurons oscillate at about 50 Hz (b). At 1.0 s, 1.2 s, and 1.4 s, the first HVC_RA_ neuron is stimulated with a DC of 12 mA to simulate song neural activity. During the song, HVC_RA_ neurons fire sparsely, and RA projection neurons show burst firing. Besides, it takes almost 65 ms for RA neurons to return to the oscillatory state.

Before studying the AFP regulation function, we show how our model generates syllables, the smallest units of birdsong. Each syllable in real birds usually lasts 10 to 100 milliseconds with its fundamental frequency between 3 and 6 kHz. Its production only involves song premotor nuclei, HVC and RA, and syrinx. In order to produce a single syllable, we set each element in a synaptic conductance matrix *g*_HR−RP×_*Г*_HVC−RA_ between [0, *g*_HR−RP_] at random, as shown in Figure 4(a). Under this condition, RA projection neurons were activated by HVC to show burst firing and produce a corresponding syllable without the impact from AFP, which can be seen in Figures 4(b) and 5.

HVC_RA_ neurons were in the polarization state and RA projection neurons oscillated at about 50 Hz before the song. At 1.0 s, 1.2 s, and 1.4 s, we stimulated the first HVC_RA_ neuron with a DC current of 12 mA and the first HVC_X_ neuron with a DC of 18 mA. During the song, HVC_RA_ neurons fired sparsely, and RA projection neurons showed burst firing. Besides, it took almost 65 ms for RA neurons to return to the oscillatory state. Compared with the *j*th HVC_RA_ neuron, the activity of the *j* + 1th neuron began later and lasted shorter.

### 5.2. The Activity and Features of AFP

**Figure 6:**
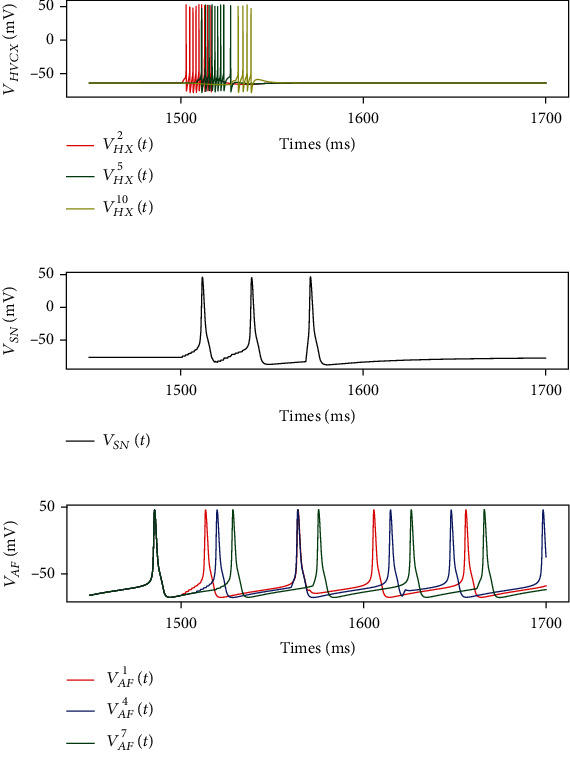
Membrane voltages of neurons in HVC and Area X. Before the song, HVC_X_ neurons (a) and SN neuron (b) are in the polarization state while AF neurons oscillate at about 20 Hz (c). At 1.5 s, the first HVC_X_ neuron is stimulated with a DC of 15 mA to simulate AFP neural activity.

During both tutoring and singing, AFP adjusts *Г*_HR−RP_ through its delayed input to RA based on its intrinsic activities. The activities of HVC_X_ neurons and Area X neurons are shown in Figure 6 with matrices *Г*_HX−AF_, *Г*_AF−DP_, *Г*_DP−LMAN_, and *Г*_LMAN−RA_ set as identity matrices. Constructed in the form of feed-forward neural circuits, HVC_X_ neurons showed similar patterns with HVC_RA_ neurons. In Area X, the SN neuron was in the polarization state, and AF neurons oscillated at about 20 Hz disorderly until the occurrence of activation. Once HVC neurons were stimulated at 1.5 s, SN neurons began to fire at 30 Hz. At the same time, AF neurons “refreshed” waiting time and fired sequentially. As time goes on, they gradually returned to fire disorderly.

**Figure 7:**
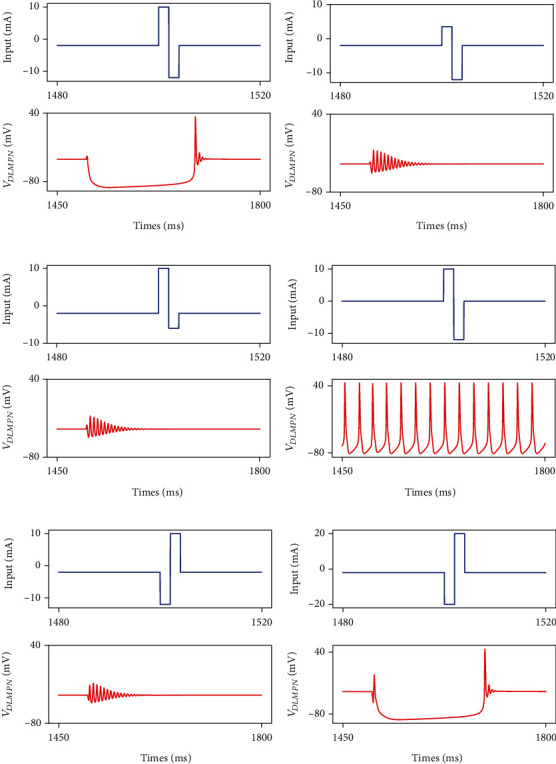
The activities of a single DLM projection neuron under different currents. (a) *I*_nor_ = −2 mA, *I*_pos_ = 10 mA, and *I*_neg_ = −12 mA. (b) *I*_nor_ = −2 mA, *I*_pos_ = 2 mA, and *I*_neg_ = −12 mA. (c) *I*_nor_ = −2 mA, *I*_pos_ = 10 mA, and *I*_neg_ = −5 mA. (d) *I*_nor_ = 0 mA, *I*_pos_ = 10 mA, and *I*_neg_ = −12 mA. (e) *I*_nor_ = −2 mA, *I*_pos_ = 10 mA, and *I*_neg_ = −12 mA. (f) *I*_nor_ = −2 mA, *I*_pos_ = 20 mA, and *I*_neg_ = −20 mA. *I*_pos_, *I*_neg_, and *I*_nor_ represent three types of currents, with *I*_pos_ and *I*_neg_ both lasting 2 ms while *I*_nor_ lasting the other time.

Including *I*_*T*_ and *I*_*h*_ currents, DLM projection neurons have more complex properties than general neurons based on the HH model. To understand the activity of DLM projection neurons, we use different DC currents to stimulate a single DLM projection neuron, as shown in Figure 7. The stimulation current contains 3 key values: normal current *I*_nor_, positive current *I*_pos_, and negative current *I*_neg_. *I*_pos_ and *I*_neg_ both lasted 2 ms while *I*_nor_ was maintained at another time. In Figure 7(a), under the condition where *I*_nor_ = −2 mA, *I*_pos_ = 10 mA, and *I*_neg_ = −12 mA, the DLM projection neuron fired 157 ms later than the stimulation time. Nevertheless, when *I*_pos_ or *I*_neg_ are much less than 10 mA, the neuron fired immediately and stabilized gradually, as shown in Figures 7(b) and 7(c). When the normal current *I*_nor_ is 0, the DLM projection neuron fired at 40 Hz without global inhibition, and its equilibrium state was hardly disrupted by *I*_pos_ and *I*_neg_ (see Figure 7(d)). Figures 7(e) and 7(f) discuss the effect of reversing *I*_pos_ and *I*_neg_. With order reversed and currents unchanged, the neuron fired immediately (see Figure 7(e)). However, with currents sufficiently large, the neuron has shown delayed activity again (see Figure 7(f)).

**Figure 8:**
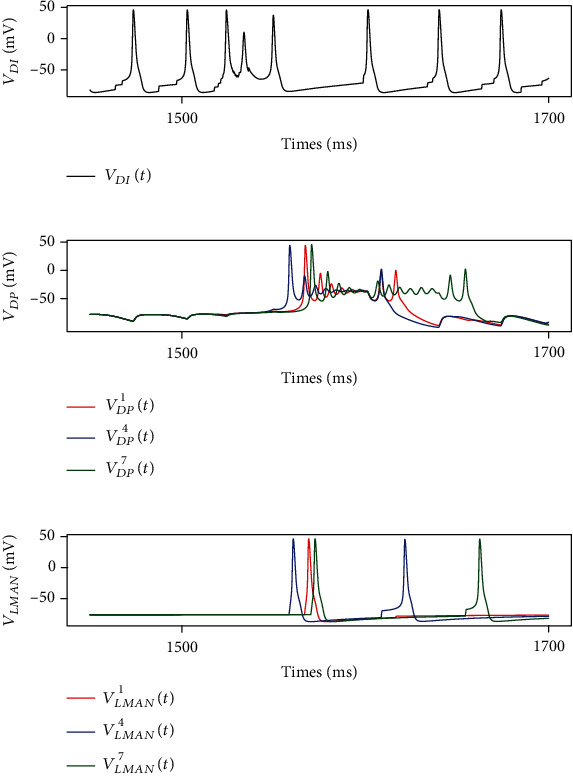
Membrane voltages of neurons in DLM and LMAN. Before the song, DLM_IN_ neurons fire at about 30 Hz (a) while DLM_PN_ neurons (b) and LMAN neurons (c) are in the polarization state. At 1.5 s, the first HVC_X_ neuron is stimulated with the DC of 15 mA. However, DLM_PN_ neurons do not fire until 1.57 s. Besides, LMAN neurons process the input from DLM_PN_ and send them to RA.

Figure 8 shows the activity of DLM and LMAN. Before the song, DLM projection neurons were in the polarization state while inhibitory neurons oscillated at about 30 Hz. Once activated, DLM projection neurons have shown complex firing patterns with their activities delayed by 50-100 ms. LMAN neurons processed the input from DLM projection neurons and stimulated RA 50-100 ms later than HVC_RA_ neurons. The time difference between the inputs from AFP and HVC helps birds learn and maintain their song.

### 5.3. Song Learning

The specific role of AFP in song learning has been fully shown in studies on zebra finch disease. Although AFP lesions have no obvious destructive effect in healthy adults [23], they can prevent the normal progress of song learning in young birds, leading to songs with highly abnormal features [5]. This shows that AFP plays a key role in song learning by adjusting to neural connectivity in song premotor pathways.

**Figure 9:**
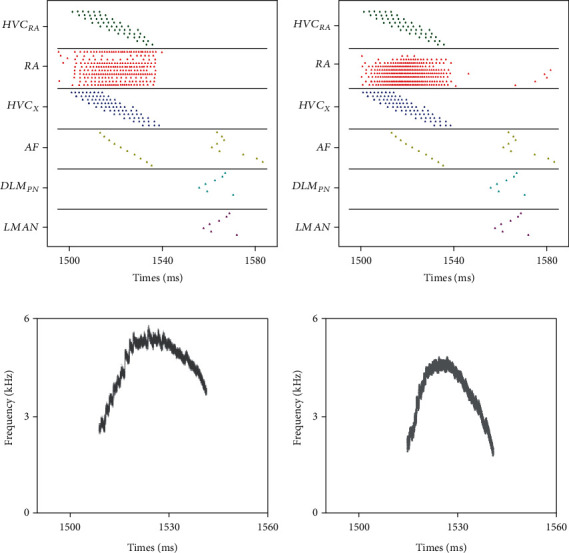
The raster plots and syllables at the first trial and 1000th trial. (a) and (b) show birdsong neural circuit activities before and after training, respectively, where each point represents a spike. (c) and (d) are the corresponding syllables with a basic frequency between 2 and 6 kHz.

To study AFP's role during song learning, we embedded the AFP model to the song premotor pathway. The AFP structure was assumed to integrate already the auditory feedback information, which would be considered in the discussion. The first HVC_RA_ and HVC_X_ neurons were activated every 1.5 s for 1000 times to simulate song learning. The raster plots and syllables before and after training are shown in Figure 9. The activity pattern of RA neurons changed significantly due to the adjustment caused by AFP. Compared to the initial state, RA neurons controlling *T*(*t*) were less active while those controlling *P*(*t*) were more active, resulting in songs with a lower frequency and longer duration.

**Figure 10:**
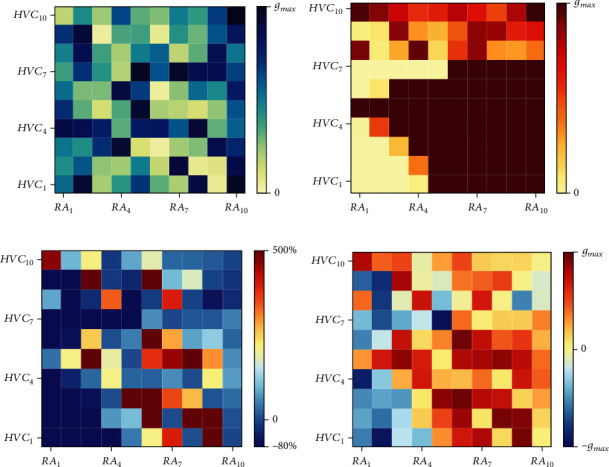
Synaptic conductance matrix and its adjustment caused by AFP during song learning. (a) and (b) show synaptic conductance matrices before and after training with color depth representing conductance intensity. (c) and (d) show adjustment rate and adjustment amount of synaptic conductance between each HVC_RA_ neuron and each RA neuron after training. In each subgraph, the square in (*i*, *j*) represents the synaptic conductance from HVC_i_ to RA_j_ with its color indicating the value.

AFP's adjustments to the structure of the song premotor pathway and the corresponding syllable are significant. For further understanding, we show two stages of the synaptic conductance matrix from HVC to RA and its change process on adjustment amount and adjustment rate in Figure 10. It can be clearly seen that the connectivity between HVC and RA in Figure 10(a) is randomly distributed. Simultaneously, in Figure 10(b), it has an obvious distribution characteristic, which corresponds to the syllable feature.  Figure 10(c) shows each synaptic conductance adjustment rate from an increase of 500% to a decrease of 80%, and Figure 10(d) shows the adjustment amount from *g*_max_ to −*g*_max_ (*g*_max_ = *g*_HR−RP_). The patterns in Figure 10(c) and Figure 10(d) were mainly controlled by AFP's neural structure, which was assumed to integrate feedback information. Activated by HVC, AFP could adjust the activity of RA and the features of syllables, constructing a basic song learning process for birds.

**Figure 11:**
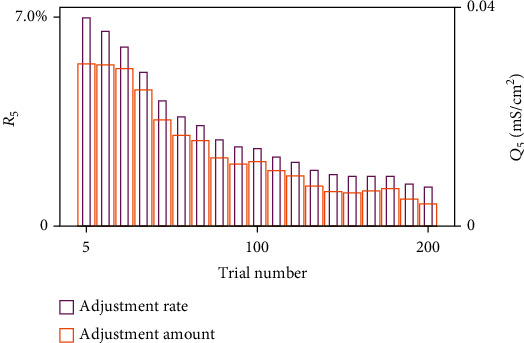
The mean adjustment of AFP every 5 trials during song learning. For five trials, synaptic conductance was maximally adjusted with a growth rate of 7%, an increase of 0.04 mS/cm^2^. With an increasing number of trials, adjustment amount *Q*_5_ and rate *R*_5_ both go down.

Figure 11 shows the changes in the conductance between HVC and RA neurons every 5 trials, including adjustment rate *R*_5_ and adjustment amount *Q*_5_, whose formulas are

(31) $$\begin{array}{c} \begin{array}{c} Q_{5}\left(n\right)=\frac{\sum_{j=1}^{10}\sum_{i=1}^{10}\left|g_{ij}\left(n+5\right)-g_{ij}\left(n\right)\right|}{100},\\ R_{5}\left(n\right)=\frac{\sum_{j=1}^{10}\sum_{i=1}^{10}\left|g_{ij}\left(n+5\right)-g_{ij}\left(n\right)\right|/g_{ij}\left(n\right)}{100}, \end{array} \end{array}$$

where *g*_ij_(*n*) represents the conductance from HVC_i_ to RA_j_ during the *n*th trial. Statistics show that the adjustment rate *R*_5_ and amount *Q*_5_ decreased with an increasing number of trials. The highest adjustment rate for five trials is 7%, with an increase of 0.04 mS/cm^2^. These figures show the specific impact of AFP during song learning. We would then study what AFP does when an adult bird gets deafen and what the differences between the two processes are.

### 5.4. Auditory Feedback Distortion

Although AFP lesions have no obvious destructive effect on healthy adult birds, they can prevent song degradation caused by deafening. Experiments have found that auditory feedback distortion occurs when birds get deaf with synapses on HVC_X_ neurons weakened [5] and song disrupted. Nevertheless, LMAN damage can prevent these results [24], and inactivating LMAN can reverse the syllable changes caused by DAF [13, 24]. Here, our model is used to study the distortion process of deaf birds and the impact of LMAN damage.

**Figure 12:**
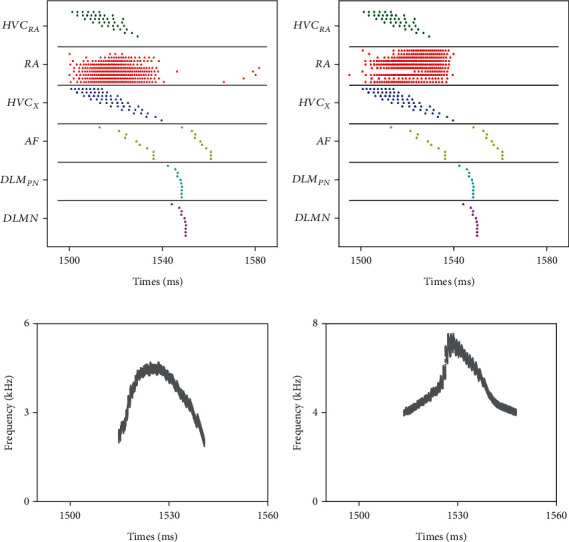
The raster plots and syllables at the first trial and 200th trial. (a) and (b) show birdsong neural circuit activities before and after training, respectively, where each point represents a spike. (c) and (d) are the corresponding syllables with a basic frequency between 2 and 8 kHz.

Due to the experimental observation that synapses of HVC_X_ cells would get gradually weak once birds get deaf [5], we reduced maximum synaptic conductance between HVC_X_ neurons into *g*_HX−HX_/2 to simulate this process. The neural circuit structure after song learning in Figure 10(b) was assumed as healthy adults' structure with syllables crystallized and initial condition during DAF. Compared with the initial condition (see Figures 12(a) and 12(c)), raster plots and syllables show evident change after 200 trials affected by deafening (see Figures 12(b) and 12(d)). The RA activity adjustment led to syllables with higher basic frequency (the highest is close to 8 kHz) and a longer duration (starting moment remained unchanged while ending moment extended).

**Figure 13:**
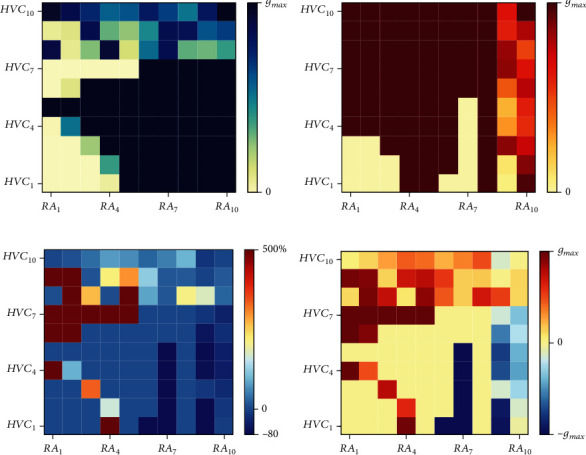
Synaptic conductance matrix and its adjustment caused by deafening during DAF. (a) and (b) show synaptic conductance matrices before and after training with color depth representing conductance intensity. (c) and (d) are adjustment rate and adjustment amount caused by AFP. (c) and (d) show adjustment rate and adjustment amount of synaptic conductance after training. In each subgraph, the square in (*i*, *j*) represents the synaptic conductance from HVC_i_ to RA_j_ with its color indicating the value.

Besides, we analyzed the synaptic conductance matrix and its adjustment caused by deafening during DAF in Figure 13. The connectivity pattern between HVC and RA in Figure 13(a) has been changed into another in Figure 13(b) by AFP under weak synapses between HVC_X_ cells.  Figure 13(c) shows each synaptic conductance adjustment rate from 500% to -80%, and Figure 13(d) shows the adjustment amount from *g*_max_ to −*g*_max_ (*g*_max_ = *g*_HR−RP_). The patterns of adjustment rate and amount correspond to the regulation of AFP with HVC_X_ synapses weakened.

**Figure 14:**
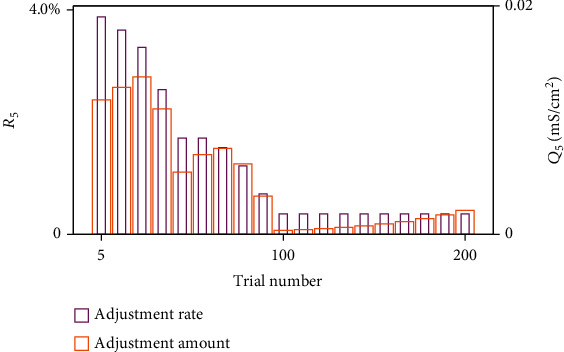
The mean adjustment of AFP every 5 trials during DAF. The highest adjustment rate is 4% for five trials, and the highest adjustment amount is 0.02 mS/cm^2^. With an increasing number of trials, *Q*_5_ and *R*_5_ broadly go down, but they get stable or even increase during some trials.

Different from those during song learning, *R*_5_ and *Q*_5_ during DAF had two states. Although they broadly went down, they got stable or even increased during some trials. As shown in Figure 14, R_5_ decreased during the first 20 trials and remained stable between 20th and 30th trials. *Q*_5_ went down generally, but it remained stable or even increased in some trials, such as those after the 100th trial. On the other hand, lesions of LMAN could prevent the adjustment through weakening HVC_X_ synapses, proving that if the adult bird with LMAN damaged gets deaf suddenly, there would be no syllable distortion.

**Figure 15:**
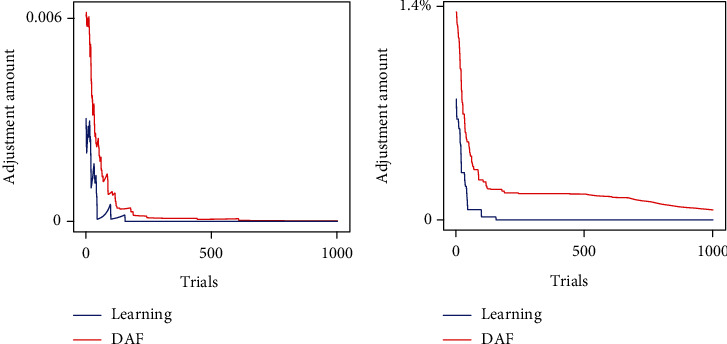
AFP's adjustment amount (a) and rate (b) for each trial during song learning and DAF. They have two phases: normal phases where the adjustment decreases with an increasing number of trials and abnormal phases where the adjustment remains stable or even increases.

Figure 15 shows the curve of average adjustment amount $\bar{Q}\left(n\right)$ and the average adjustment rate $\bar{R}\left(n\right)$ of HVC-RA conductance during song learning and DAF. The corresponding formulas are

(32) $$\begin{array}{c} \begin{array}{c} \bar{Q}\left(n\right)=\frac{\sum_{j=1}^{10}\sum_{i=1}^{10}\left|g_{ij}\left(n+1\right)-g_{ij}\left(n\right)\right|}{100},\\ \bar{R}\left(n\right)=\frac{\sum_{j=1}^{10}\sum_{i=1}^{10}\left|g_{ij}\left(n+1\right)-g_{ij}\left(n\right)\right|/g_{ij}\left(n\right)}{100}, \end{array} \end{array}$$

where *g*_ij_(*n*) represents the conductance from HVC_i_ to RA_j_ during the *n*th trial.

The average adjustment rate curve under two conditions was monotonic and nonincreasing. In contrast, the adjustment amount curve fluctuated above and below, especially during DAF, indicating that AFP's adjustment is phased. For example, the adjustment amount decreased in the first 46 trials, while it remained stable or even increases in some special trials during the DAF. Besides, compared to that during song learning, the adjustment process of AFP is stronger and shorter.

## 6. Discussion

Our study replicated and extended previous research by Henry et al. on birdsong neural circuits. They constructed a physiological model of song premotor pathway and the syrinx, showing the neural process of song production [11]. In the same year, they also found that the intrinsic circuit within RA may greatly influence the features of birdsong [9]. However, they did not consider AFP's adjustment to song premotor pathway and syllables. Despite many physiological experiments on AFP, its modeling studies are limited to describe a single neuron [9] and unable to support its role during song learning and DAF. Due to urgent research needs for AFP's modeling study, we constructed an AFP-mediated birdsong neural model to show that AFP could significantly affect syllables based on its adjustment to the structure of the song premotor pathway. Through qualitative and quantitative analysis of AFP's adjustment, we found the adjustment amount increased with more trials in some special cases, countering our initial expectations that AFP's adjustment gradually weakened in prolonged trains. Our findings need experimental verifications and hold the potential to advance the field of birdsong neural modeling research.

In our model, we set HVC as a sparse signal generator and AFP as a song-regulated setup, which are both thought to integrate auditory feedback already. However, we did not show the specific processes. In actual song learning, the auditory feedback is variable and sent to HVC and AFP from other nervous tissue, such as dopamine (DA) neurons in the ventral tegmental area [25]. These processes need to be addressed to achieve a deeper understanding of learning and memory in our model.

Our model for song production has not been complete. For example, the interface core (NIf) is thought to control the order of syllables through its input to HVC [26]. The model for NIF needs to be built to expand our syllables into a complete birdsong. Besides, a new connection from AFP to HVC is being recognized since a finding by Hamaguchi et al. that destroying LMAN can prevent the weakening of HVC_X_ synapses due to deafness [5]. Therefore, birdsong modeling should consider a bidirectional connection between HVC and AFP, which may play an important role during song learning and DAF. Finally, all neurons in our model are represented by the HH model, which may be too complex for large-scale modeling. These neurons should be constructed on a simplified model, such as the FitzHugh-Nagumo model, for larger simulation.

## 7. Conclusions

In this study, we proposed an AFP-mediated premotor pathway model of birdsong. The model successfully simulated the learning process in young birds and the syllable distortion process in deaf birds. These processes were based on the following three aspects. Firstly, RA neurons acted under a certain HVC-RA conductance matrix and transmitted structure information of the song premotor pathway to the syrinx, which then integrated input and generated syllables corresponding to the structure. Secondly, AFP that had integrated feedback information was active during birdsong and sent delayed input to RA, resulting in an adjustment to the conductance structure from HVC to RA and eventually regulating syllables. These simple processes were repeated to form the learning process of young birds. Thirdly, even with the same AFP structure, the weakening of synapses in HVC_X_ neurons due to deafening would indirectly lead to the rapid syllable distortion process through AFP. The key point of the distortion process was that the weakened HVC_X_ input caused abnormal activities of DLM projection neurons and then affected the input from LMAN to RA. The altered input disturbed the conductance of the song premotor pathway, causing syllable distortion in deaf birds. The syllable regulation's neural mechanisms explain many experimental phenomena about AFP, and the phased regulation of AFP we found promotes the study of vocal learning and maintenance.

## Data Availability

The data used to support the findings of this study are included within the article.

## References

[1] Nottebohm F., Paton J. A., Kelley D. B. (1982). Connections of vocal control nuclei in the canary telencephalon. *Journal of Comparative Neurology*.

[2] Doupe A. J., Kuhl P. K. (1999). Birdsong and human speech: common themes and mechanisms. *Annual Review of Neuroscience*.

[3] Brainard M. S., Doupe A. J. (2002). What songbirds teach us about learning. *Nature*.

[4] Brainard M. S. (2004). Contributions of the anterior forebrain pathway to vocal plasticity. *Annals of the New York Academy of Sciences*.

[5] Hamaguchi K., Tschida K. A., Yoon I., Donald B. R., Mooney R. (2014). Auditory synapses to song premotor neurons are gated off during vocalization in zebra finches. *Elife*.

[6] Hahnloser R. H. R., Kozhevnikov A. A., Fee M. S. (2002). An ultra-sparse code underlies the generation of neural sequences in a songbird. *Nature*.

[7] Mooney R., Rosen M., Sturdy C. (2002). A bird’s eye view: top down intracellular analyses of auditory selectivity for learned vocalizations. *Journal of Comparative Physiology A: Sensory, Neural, and Behavioral Physiology*.

[8] Kojima S., Doupe A. J. (2007). Song selectivity in the pallial-basal ganglia song circuit of zebra finches raised without tutor song exposure. *Journal of Neurophysiology*.

[9] Abarbanel H. D. I., Gibb L., Mindlin G. B., Talathi S. (2004). Mapping neural architectures onto acoustic features of birdsong. *Journal of Neurophysiology*.

[10] Tschida K. A., Mooney R. (2012). Deafening drives cell-type-specific changes to dendritic spines in a sensorimotor nucleus important to learned vocalizations. *Neuron*.

[11] Abarbanel H. D. I., Talathi S. S., Mindlin G., Rabinovich M., Gibb L. (2004). Dynamical model of birdsong maintenance and control. *Physical Review E*.

[12] Peng Z., Zhang X. B., Liu Y. (2013). Ultrastructural and electrophysiological analysis of Area X in the untutored and deafened Bengalese finch in relation to normally reared birds. *Brain Research*.

[13] Andalman A. S., Fee M. S. (2009). A basal ganglia-forebrain circuit in the songbird biases motor output to avoid vocal errors. *Proceedings of the National Academy of Sciences*.

[14] Fee M. S., Kozhevnikov A. A., Hahnloser R. H. R. (2004). Neural mechanisms of vocal sequence generation in the songbird. *Annals of the New York Academy of Sciences*.

[15] Spiro J. E., Dalva M. B., Mooney R. (1999). Long-range inhibition within the zebra finch song nucleus RA can coordinate the firing of multiple projection neurons. *Journal of Neurophysiology*.

[16] Chi Z., Margoliash D. (2001). Temporal precision and temporal drift in brain and behavior of zebra finch song. *Neuron*.

[17] Rossant C., Kadir S. N., Goodman D. F. M. (2016). Spike sorting for large, dense electrode arrays. *Nature Neuroscience*.

[18] Benezra S. E., Narayanan R. T., Egger R., Oberlaender M., Long M. A. (2018). Morphological characterization of HVC projection neurons in the zebra finch (Taeniopygia guttata). *Journal of Comparative Neurology*.

[19] Scharff C., Nottebohm F. (1991). A comparative study of the behavioral deficits following lesions of various parts of the zebra finch song system: implications for vocal learning. *Journal of Neuroscience*.

[20] Farries M. A., Perkel D. J. (2002). A telencephalic nucleus essential for song learning contains neurons with physiological characteristics of both striatum and globus pallidus. *Journal of Neuroscience*.

[21] Abarbanel H. D. I., Gibb L., Mindlin G. B., Rabinovich M. I., Talathi S. (2004). Spike timing and synaptic plasticity in the premotor pathway of birdsong. *Biological Cybernetics*.

[22] Laje R., Gardner T. J., Mindlin G. B. (2002). Neuromuscular control of vocalizations in birdsong: a model. *Physical Review E*.

[23] Bottjer S. W., Miesner E. A., Arnold A. P. (1984). Forebrain lesions disrupt development but not maintenance of song in passerine birds. *Science*.

[24] Warren T. L., Tumer E. C., Charlesworth J. D., Brainard M. S. (2011). Mechanisms and time course of vocal learning and consolidation in the adult songbird. *Journal of Neurophysiology*.

[25] REINER A., Perkel D. J., Mello C. V., Jarvis E. D. (2004). Songbirds and the revised avian brain nomenclature. *Annals of the New York Academy of Sciences*.

[26] Hosino T., Okanoya K. (2000). Lesion of a higher-order song nucleus disrupts phrase level complexity in Bengalese finches. *Neuroreport*.

